# Greater Engagement with Health Information Is Associated with Adoption and Maintenance of Healthy Lifestyle Behaviours in People with MS

**DOI:** 10.3390/ijerph17165935

**Published:** 2020-08-15

**Authors:** Xin Lin, Maggie Yu, George A. Jelinek, Steve Simpson-Yap, Sandra Neate, Nupur Nag

**Affiliations:** 1 Neuroepidemiology Unit, Centre for Epidemiology and Biostatistics, Melbourne School of Population and Global Health, The University of Melbourne, Melbourne, VIC 3010, Australia; xin.lin2@unimelb.edu.au (X.L.); maggie.yu@unimelb.edu.au (M.Y.); g.jelinek@unimelb.edu.au (G.A.J.); steve.simpsonyap@unimelb.edu.au (S.S.-Y.); sandra.neate@unimelb.edu.au (S.N.); 2 Menzies Institute for Medical Research, University of Tasmania, Hobart, TAS 7000, Australia

**Keywords:** health communication, behavioural change, multiple sclerosis, lifestyle, longitudinal observational study

## Abstract

Health communication offers an important means for patients to make informed decisions for illness self-management. We assessed how the level of engagement with selected health information at baseline is associated with the adoption and maintenance of lifestyle behaviours at a 5-year follow-up in people with multiple sclerosis (MS). Non-engagers were compared to engagers of information delivered online and print (medium), and with engagers who additionally attended a live-in workshop (high). Engagement was assessed against lifestyle behaviours by log-binomial regression. Information engagers had higher education, and were less likely to have severe disability, clinically significant fatigue, or obesity. Medium and high baseline engagement was associated with adopting healthy behaviours for omega 3 supplementation (RR = 1.70; 95%CI: 1.02–2.84), physical activity (RR = 2.16; 95%CI: 1.03–4.55), and dairy non-consumption (RR = 3.98; 95%CI: 1.85–8.56) at 5 years; associations were stronger among high engagers. Only high baseline engagement was associated with maintaining behaviours from baseline to 5 years, specifically for omega-3 (RR = 1.26; 95%CI: 1.06–1.49) and vitamin D supplementation (RR = 1.26; 95%CI: 1.04–1.54) and dairy non-consumption (RR = 1.47; 95%CI: 1.03–2.10). Health communication that includes face-to-face information delivery and practical tools for implementation in daily living may be optimal for adopting and maintaining lifestyle behaviours in people with MS.

## 1. Introduction

Multiple sclerosis (MS) is a progressive autoimmune neurological disorder, which manifests in a diverse range of cognitive, physical, and psychological symptoms, that can impact upon daily living and reduce health-related quality of life [1,2,3]. While the precise aetiology is unknown, MS appears to result from a complex interaction of genetic predisposition, early life exposure to certain viral infections, and environmental factors, including smoking, childhood obesity, low sun exposure, and low fish/polyunsaturated fatty acid intake [4,5]. Some of these environmental risk factors have also been associated with disease progression. However, inconsistent results across studies, and the absence of randomised control trials, limit their interpretation and translation for effectiveness for disease prevention and treatment. Nevertheless, many people with MS (pwMS) undertake lifestyle modification to assist in disease management [6], either alone or in combination with drug therapy.

In the context of secondary prevention and self-management of chronic illnesses through lifestyle modifications, health communication can play a role in facilitating behavioural change. Health communication refers to the communication of health information to patients via multiple channels, including online, print, and individual and group interventions. Health communication, which provides updated information, relevant resources, and empowers patients through targeted and tailored skills and strategies, may increase participation in health behaviours [7,8,9].

Face-to-face interventions in individual and group settings are traditional approaches to inform patients that have been shown to be effective in conveying information to promote health behaviours for pwMS and other chronic conditions [10]. Online health information is regarded as a convenient and low-cost complementary resource for symptom management and behaviour changes [11]. Survey responses from over 8000 pwMS in the USA revealed that, although physicians remain the most trusted source of health information, the internet was the first source for more than 85% of respondents [12]. Although the reliability, adequacy, and relevance of online information remain challenging, pwMS in general find rapidly updated online information useful in helping cope with symptoms and being more active in health decision-making [13]. Patient-centred workshops effectively increase self-efficacy, which ultimately stimulate behavioural change [14,15]. PwMS reported that their confidence and knowledge improved through experiential learning and daily living skills practice, both enablers for adherence to lifestyle behaviours [16].

We propose pwMS who engage with health information are more likely to modify their health behaviours, and assess this through analysis of data extracted from the Health Outcomes and Lifestyle In a Sample of people with Multiple sclerosis (HOLISM) study. This is an international longitudinal observational study comprising of self-reported lifestyle, socio-demographic, and health status data on pwMS [17,18]. Also queried is participant engagement with select information resources promoting healthy lifestyle behaviours—a book recommending lifestyle behaviours based on scientific evidence [19]; a website communicating the recommendations from the book, including recipes and guided meditation to aid in incorporating lifestyle behaviours into daily life; and live-in workshops providing interactive sessions delivered by health professionals, and a program of wellbeing and support, including daily meditation, cooking demonstrations, and shared storytelling [14,20].

We previously showed that pwMS who engaged with health information delivered by either this book, website, or workshops were associated with better health-related quality of life and lower rates of depression and fatigue risk [20]. Engagement with all three forms of information resources had the strongest associations with positive health outcomes; these outcomes may be associated with changed behaviours. We thus sought to assess whether pwMS who engaged with health information resources changed lifestyle behaviours differently from participants who did not. Understanding how health communication impacts behavioural change may enable informed decision-making for implementation of self-management strategies for pwMS.

## 2. Materials and Methods

### 2.1. Study Design and Participants

PwMS were invited to participate in the continuing HOLISM longitudinal observational study as described previously [17]. In brief, pwMS aged ≥ 18 years were recruited via online platforms relating to MS in 2012, and consenting participants invited to complete an online survey via SurveyMonkey^®^ at 2.5-year intervals. The 100-question survey captures self-reported data on socio-demographics, and clinical, lifestyle, and health outcomes. The study was approved by the Health Sciences Human Ethics Subcommittee at The University of Melbourne (HESC 1545102). At baseline, 2466 participants self-reporting a clinician-confirmed diagnosis of MS completed the survey; of whom, 952 (38.6%) completed both baseline and 5-year surveys and were included in our analysis.

### 2.2. Data Collection and Measurement

#### 2.2.1. Demographics

A range of demographic factors, including age, sex, level of education, employment, and marital status, were captured. Age was calculated from the difference between the reported date of birth and the date of survey completion and categorised to the interquartile year range. Sex (male, female), highest level of education (no formal schooling, primary school, secondary school, vocational training, graduate degree, post-graduate degree), employment status (work full-time, work part-time, self-employed, unspecified paid work, stay at home parent/carer, full-time student, unemployed, seeking/not seeking employment, retired due to age/medical reasons/disability, work status not specified), and marital status (married, cohabiting/partnered, separated/divorced/widower, single) were queried at each review; the latter three variables were re-categorised as indicated in the tables.

#### 2.2.2. Clinical Variables

Researcher-devised items assessed the self-reported type of MS diagnosed and duration since onset in years. MS type was grouped into relapsing-remitting (RR)MS/benign and progressive (secondary progressive (SP)MS/primary progressive (PP)MS/progressive relapsing (PR)MS), and duration since MS onset was categorised into quartiles. Disability status was measured using the Patient-Determined Disease Steps (PDDS), a self-reported measure of ambulatory disability, which has been cross-validated against the Expanded Disability Status Scale (EDSS) [21,22]. The PDDS is scored ordinally from 0 (normal) to 8 (bed-bound). We categorised the validated survey scores into three groups for analyses: Low disability (no walking impairment) = 0–2, moderate disability (gait disability/single cane) = 3–5, severe disability (requirement for two canes/wheelchair/bed) = 6–8. Fatigue was measured by the 9-item Fatigue Severity Scale (FSS), which queries 9 statements each with a 7-point Likert scale from disagree = 1 to agree = 7. A mean total score > 5 denotes clinically significant fatigue [23]. Body mass index (BMI) was calculated from participant-reported height (m) and weight (kg) using the function weight/height^2^, and classified as underweight/normal, overweight, and obese according to World Health Organisation classifications [24].

#### 2.2.3. Health Communication

The information source for lifestyle modification was queried by the frequency of visits (selection from a 6-option drop-down menu from rarely to daily) to the website http://www.overcomingms.org, a binary no/yes response to have read the book “*Overcoming Multiple Sclerosis*” [25] and attendance at a live-in workshop based on these resources. We chose to use a hierarchical categorisation reflecting increasing engagement with these three sources of health information: 1. None—participants responding “no” to all of website visits, book and workshop; 2. Medium—participants visiting the website and/or yes to having read the book; and 3. High—attendance at a live-in workshop and satisfying medium participant criteria.

#### 2.2.4. Lifestyle Behaviours

Participant self-reported consumption of omega-3 supplements was queried as a binary no/yes response. Vitamin D was measured based on self-reported IU dose and frequency, from which the average daily dose was estimated. A minimum of 5000 IU/day of vitamin D supplementation, as recommended by health information resources, was used as a cut off for data analysis. Participants were also asked whether they intentionally exposed themselves to the sun to increase their vitamin D level (no/yes). Two binary variables of whether participants consumed dairy and meat were derived from responses to the items within the modified Diet Habits Questionnaire (DHQ) [17,26]. Participants who responded, *‘I do not consume dairy’* to the question *“When having dairy, how often use reduce/low-fat products?”* were considered dairy free. For meat consumption, participants were asked *“How often do you eat processed meat?”* and *“How often do you trim visible fat/remove skin from meat/poultry before cooking?”* A dichotomized variable (0 = do not consume meat; 1 = consume meat) was created based on responses of *“I do not eat meat”* to either question. Current smoking status was queried and dichotomised as current versus never/ex-smoker. Two researcher-devised items measured the frequency and duration of meditation practice in the last 12 months. For analyses, we collapsed two items and created a binary variable of meditation (1 = meditated ≥ 10 min/week vs. 0 = less frequently). Physical activity was assessed using the International Physical Activity Questionnaire-Short Form (IPAQ-SF); a validated 7-day recall of frequency and duration of walking, and moderate- and vigorous-intensity activities [27]. For analysis, a binary variable for vigorous physical activity ≥ 30 min/day ≥ 3 times/week was derived.

### 2.3. Statistical Analysis

All analyses were conducted in Stata version 14.2 (StataCorp, College Station, TX, USA) with the statistical significance threshold set at *p* < 0.05. Log-binomial regression models were used to evaluate associations of the health communication mode with lifestyle behaviours at baseline and a 5-year follow-up, estimating prevalence ratios (PRs). Changes in lifestyle behaviours were analysed using dichotomous outcome variables created according to the difference in reported lifestyle behaviours at baseline and 5-year reviews. Results were presented as risk ratios (RRs). All multivariable models were adjusted for age, sex, education, duration since MS onset, employment status, clinically significant fatigue, and disability at baseline.

#### Adoption and Maintenance of Lifestyle Behaviours

We assessed whether the level of resource engagement at baseline was associated with health behaviours at a five-year follow-up. Information resources recommend the following lifestyle behaviours: Consume omega-3 supplement, consume ≥ 5000 IU/day vitamin D supplement, seek sun exposure to increase vitamin D levels, undertake vigorous activity for ≥ 30 min/day ≥ 3 times/week, meditate ≥ 10 min/week, no dairy and meat consumption, and abstinence from smoking.

Binary variables (no/yes) were created based on participants’ health behaviours at both time points (Table 1) and the following associations determined:

*Adoption of health behaviours*: Participants who changed from “no” at baseline to “yes” at 5 years for omega-3 and vitamin D supplementation, sun exposure, meditation, and physical activity were considered to have adopted healthy behaviours, and were compared to the reference group of participants who were “no” at both time-points. For smoking and dairy and meat consumption, participants who changed from “yes” to “no” were considered to have adopted healthy behaviours and were compared to those who were “yes” at both time-points.

*Maintenance of health behaviours*: Participants who remained “yes” at both time-points for omega-3 and vitamin D supplementation, sun exposure, meditation, and physical activity were classified as maintaining healthy behaviours and were compared to the reference group of participants who changed from “yes” at baseline to “no” at 5 years. For smoking and dairy and meat consumption, participants who remained “no” at both time-points were compared to those who changed from “no” to “yes” in order to assess the maintenance of healthy behaviours.

## 3. Results

### 3.1. Cohort Characteristics

The study cohort comprised 952 participants who completed both baseline and 5-year follow-up surveys. Compared to participants who did not complete the 5-year survey, participants who did were generally well-educated, employed, married, with benign/RRMS, less severe disability, less fatigue, lower BMI, higher resource engagement, and a healthier lifestyle (consume omega-3 and vitamin D supplements, seek sun exposure to raise vitamin D, practice meditation, vigorously exercise frequently, do not smoke, and do not consume meat or dairy; Table S1). The included 952 participants had a similar age distribution across the three levels of engagement, with the mean age of mid- to late-40 s at baseline (Table 2). Benign/RRMS was the predominant MS type. Compared to non-engagers, engagers had lower proportions of female participants (88.3% none; 81.7% medium; 80.3% high), and higher proportions of university-qualified (59.1% none; 69.2% medium; 73.2% high) and married (75.0% none; 78.8% medium; 85.1% high) participants. Compared to non-engagers (23.0%), high engagers were more likely to be employed (40.4%); no difference in employment status was observed between non and medium engagers. Lower proportions of engagers were overweight (27.3% none vs. 18.2% medium) or obese (25.9% none; 13.1% medium; 9.2% high), reported clinically significant fatigue (73.0% none; 56.3% medium; 45.9% high), or had moderate (45.1% none; 27.8% medium; 32.4% high) or severe disability (9.1% none; 6.0% medium; 5.6% high). Compared to non-engagers (median = 15.4), medium engagers also had a shorter duration since MS onset (median = 10.4). At 5 years, cohort characteristics across baseline engagement groups were mostly comparable with cohort characteristics at baseline, with only employment and marital status changing to being similar across three groups (data not shown).

### 3.2. Cross-Sectional Associations

Baseline associations between resource engagement and lifestyle behaviours (Table 3) showed that, compared to non-engagers, medium-level engagers were more likely to consume omega-3 (PR = 1.42; 95%CI: 1.21–1.66) and ≥ 5000 IU/day of vitamin D (PR = 1.85; 95%CI: 1.44–2.37) supplements, seek sun exposure to raise their vitamin D (PR = 1.49; 95%CI: 1.26–1.76), and practice meditation ≥ 10 min/week (PR = 1.65; 95%CI: 1.09–2.48). These associations were stronger in high engagers (omega-3: PR = 1.56; 95%CI: 1.32–1.83; vitamin D: PR = 2.70; 95%CI: 2.09–3.49; sun exposure: PR = 1.57; 95%CI: 1.31–1.89; mediation: PR = 3.94; 95%CI: 2.60–5.97). Medium-level engagers were less likely to vigorously exercise regularly compared to non-engagers at baseline (PR = 0.58; 95%CI: 0.42–0.80). No associations were seen with physical activity and high engagers. Compared to non-engagers, medium and high engagers were less likely to be current smokers (medium: PR = 0.53; 95%CI: 0.32–0.87; high: PR = 0.12; 95%CI: 0.03–0.54), and consume dairy (medium: PR = 0.69; 95%CI: 0.62–0.78; high: PR = 0.20; 95%CI: 0.13–0.31) or meat (medium: PR = 0.67; 95%CI: 0.61–0.74; high: PR = 0.19; 95%CI: 0.13–0.28). All associations observed at baseline, except for meditation and physical activity for medium-level engagers, persisted at the 5-year follow-up (Table S2). Cross-sectionally, resource engagement was significantly associated with lifestyle behaviours at both baseline and 5-year follow-up.

### 3.3. Adopting Lifestyle Behaviours

To determine whether resource engagement at baseline could predict the adoption of healthy lifestyle behaviours at 5 years, we compared participants who adopted healthy lifestyle behaviours from baseline to 5 years to those who did not (Table 4). Of the 30% (281/952) of participants who did not consume omega-3 supplements at baseline, 33% (93/281) consumed the supplement at 5 years. Nearly half (47.5%; 453/952) of all participants did not consume ≥ 5000 IU/day vitamin D supplements at baseline, 45% (205/453) of whom consumed this daily dose at 5 years. Twenty-seven percent (257/952) of all participants did not seek out sun exposure for vitamin D at baseline, of whom 27% (70/257) adopted this behaviour at 5 years. Around 40% (391/952) of the population did not meditate for ≥ 10min/week at baseline, and 36% (140/391) adopted this behaviour at 5 years. The majority of participants (81%; 768/952) did not undertake frequent vigorous physical activity at baseline, of whom only 15% met the criteria at 5 years. Participants also stopped unhealthy lifestyle behaviours over 5 years. At baseline, 7.7% (73/952) of participants were smokers, of whom 48% (35/73) identified as non-smokers by 5 years. Half of the participants (480/952) consumed dairy at baseline, of whom 17% (81/480) reported being dairy free at 5 years. Similarly, 54.3% (517/952) consumed meat at baseline, of whom 12% followed a meat-free diet at 5 years. The adoption of healthy lifestyle behaviours varied from 12% to 48% from baseline to the 5-year follow-up. Quitting smoking and consuming vitamin D supplements achieved the highest adoption rates, while stopping meat consumption had the lowest adoption rates.

**Figure 1:**
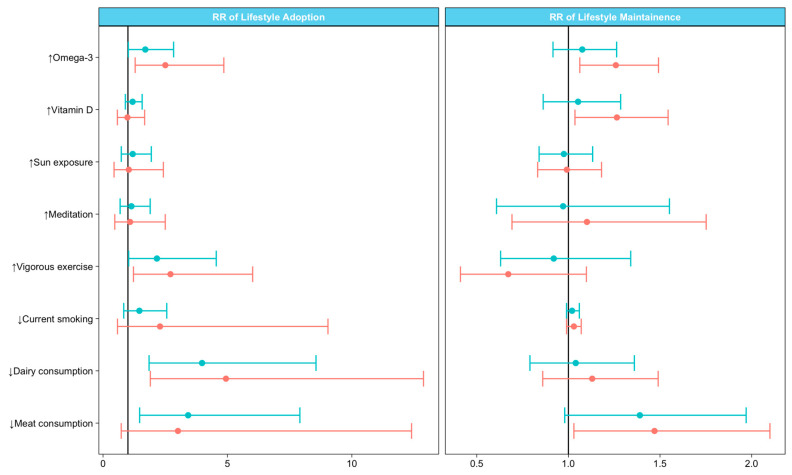
Associations of resource engagement with modifying lifestyle behaviours. Relative risk (RR) estimates for adoption and maintenance of lifestyle behaviours are illustrated by resource engagement levels (none, black with RR = 1; medium, blue; high, orange). Arrow direction indicate recommended lifestyle behaviour (upward = increase, downward = no).

The adoption of certain behaviours was associated with baseline resource engagement (Table 4, Figure 1). Compared to non-engagers, resource engagers were more likely at 5 years to consume omega-3 supplements (medium: RR = 1.70; 95%CI: 1.02–2.84; high: RR = 2.50; 95%CI: 1.29–4.85), undertake frequent vigorous physical activity (medium: RR = 2.16; 95%CI: 1.03–4.55; high: RR = 2.71; 95%CI: 1.22–6.01), and stop dairy consumption (medium: RR = 3.98; 95%CI: 1.85–8.56; high: RR = 4.94; 95%CI: 1.90–12.88); the strongest associations were seen with high engagers. Only medium-, not high-, level engagement was associated with meat non-consumption at 5 years (RR = 3.42; 95CI% = 1.47–7.91). No significant associations were found between resource engagement and change in vitamin D supplementation, sun exposure, smoker status, or meditation practice; it is possible that the participants numbers were too few.

### 3.4. Maintaining Lifestyle Behaviours

To assess whether baseline resource engagement was associated with maintaining lifestyle behaviours, we compared participants who maintained healthy behaviours from baseline to 5 years to those who did not (Table 5, Figure 1). Seventy percent (671/952) of all participants consumed omega-3 supplements at baseline, of whom 73% (493/671) maintained this at 5 years. Of 49% (471/952) of participants who consumed ≥ 5000 IU/day vitamin D supplements at baseline, 79% (372/471) maintained this dose at 5 years. More than two-thirds (638/952) of participants sought sun exposure for vitamin D at baseline, and 74% (470/638) maintained this behaviour at 5 years. Among 19% (184/952) of participants who practiced meditation ≥ 10 min/week at baseline, 56% (103/184) maintained this practice at 5 years. Of the 19% of participants (182/952) who undertook frequent vigorous physical activity at baseline, 54% (99/182) maintained this level at 5 years. Almost 90% (847/952) of participants did not smoke at baseline, nearly all (98%) of whom remained as non-smokers at 5 years. Near half of the participants did not consume dairy at baseline (431/952), and the majority (79%, 339/431) remained dairy free at 5 years. Likewise, 81% (328/403) of the 42% of participants who did not consume meat at baseline remained meat free at 5 years. Maintenance of healthy behaviours from baseline to 5 years varied from 54% (vigorous physical activity) to 98% (abstinence from smoking).

Maintenance of some lifestyle behaviours were significantly associated with high-level (but not medium) resource engagement at baseline. Compared to non-engagers, high engagers were more likely to maintain intake of omega-3 (RR = 1.26; 95%CI: 1.06–1.49) and vitamin D supplements (RR = 1.26; 95%CI: 1.04–1.54), and remain dairy free (RR = 1.47; 95%CI: 1.03–2.10) at 5 years. No material associations were observed for maintaining sun exposure, meditation practice, and frequent vigorous physical activity.

## 4. Discussion

Health communication is important for informed decision-making for patients with chronic conditions. Patients feel more empowered and are more likely to adhere to treatment recommendations when they are involved in the choices they make to manage their health. We sought to examine the relationship between engagement with reading and workshop resources for a particular patient-centred and evidence-based program promoting healthy lifestyle behaviours for pwMS, with adoption and maintenance of healthy behaviours. The three engagement levels were no engagement, engagement with resources via either website and/or book (medium level), and engagement with reading resources as well as attendance at a live-in workshop (high level).

Across the three levels of engagement, participant characteristics were generally similar, with notable differences in non-engagers having more severe disability, longer duration of diagnosis, more likely progressive MS types, and the presence of clinically significant fatigue. These differences align with prior studies showing people with more severe MS tend to have lower self-efficacy, more perceived barriers to healthy behaviours, and higher levels of depression [28,29,30], all of which adversely affect intention and engagement in health interventions [31]. In our study, non-engagers were additionally more likely to be obese, possibly reflective of unhealthy diet and inactive behaviours. Further studies on larger populations would enable determination of the impact of cohort characteristics on health information-seeking behaviours, thereby allowing targeted communication for different populations.

Cross-sectionally, baseline engagement was associated with baseline and 5-year behaviours for supplement use, diet, meditation practice, sun seeking, and smoking abstinence, with higher-level engagement reflected in stronger associations. Baseline medium-level engagement was associated with physical activity; however, these engagers were less likely to meet the recommended 30 min or more, at least 3 times a week, of vigorous physical activity. These associations confirm that engagement with health information is related to lifestyle behaviour, and thus we further assessed how engagement relates to changed behaviours.

Adoption of new healthy dietary behaviours from baseline to 5 years was associated with both levels of engagement. Engagers who did not meet recommended healthy behaviours at baseline were more likely to commence omega-3 supplementation, meet physical activity recommendations, and stop consuming dairy at the 5-year follow-up. Medium-level engagers additionally adopted a meat-free diet at 5 years. Associations were strongest in pwMS who engaged with both reading and workshop resources consistent with studies showing small group programs are effective in altering dietary behaviours [32], with interactive health promotion better for the adoption of healthy behaviours than other forms of learning [33,34,35,36].

Maintaining recommended lifestyle behaviours from baseline to the 5-year follow-up was associated with engagement with both reading and workshop resources but not with engagement with reading material alone. Delivery of health information through diverse methods, including print, web-based interventions, and face-to-face live-in workshops, are associated with changes in behaviour. The success of such interventions appears to be related to the combination of the delivery of evidence from experts and practical interventions tailored to personalised goals [13,16]. Moreover, focus groups have revealed pwMS want a variety of information from a variety of sources and that this information should be both stratified, interactive, and provided by subject experts to aid in the uptake of positive lifestyle behaviours [37,38,39]. The workshops meet these criteria and where possible utilised medically trained facilitators who themselves had been diagnosed with MS, providing expert knowledge as well as personal experience.

Long-term maintenance of behaviours was restricted to most dietary modifications: Omega-3 and vitamin D supplement use and dairy consumption. These findings align with studies showing 67–81% of pwMS use dietary supplements and 37–41% follow specific diets [40]. The maintenance of behaviours that utilised a physical component—seeking sun to increase vitamin D, meditation practice, and physical activity—were not associated with health information engagement. Similarly, other active wellness programs have shown improved self-efficacy and perceived health, but no change in physical activity, for pwMS [41,42]. PwMS are known to be less active than the general population [43,44,45], and due to symptom heterogeneity, it may be that the implementation of active behaviours requires tailored and personalised recommendations. Of the physical behaviours, only meditation practice included online guided practice videos, and daily group participation in the workshops. Education on methods to incorporate a variety of accessible and disability-inclusive daily physical behaviours were introduced to the website towards the end of the period studied. Sun- seeking behaviour may not be maintained possibly due to participants not being vitamin D deficient, achieving a healthy dose potentially through dietary supplementation, or due to public health messages discouraging sun exposure.

For individuals with chronic conditions, learning information and skills to adopt and maintain healthy lifestyle behaviours offers self-empowerment through better self-management. Both medium- and high-level engagement were associated with the adoption, and only high engagement with maintenance, of mostly dietary behaviours in pwMS. This is in line with our previous cross-sectional baseline study [20] which showed a higher level of engagement with health resources was associated with better health outcomes. However, as cost and time investment may limit attendance at workshops, providing affordable and accessible education resources that can be widely disseminated is essential. Web-based interventions are accepted by pwMS due to their easy accessibility, convenience, and low cost [46,47]. Consideration should be given to transforming the unique qualities of the live-in workshops into interactive web-based learning, delivered by health professionals for small groups to complete together, and include online support groups to mitigate attrition, as well as practical lifestyle options with consideration of sociodemographics and disease stage.

Health communication, either through reading alone or combined with attendance at a face-to-face workshop, was not associated with all health behaviours for pwMS in our study. Behavioural changes are shaped by numerous individual, social, and environmental factors. Behaviours that require more time commitments or adjustments may be especially difficult to modify. In our previous qualitative study of pwMS who attended live-in workshops 3–5 years prior, participants continued to follow dietary but not physical behaviours, stating barriers to adherence to all recommended behaviours included disease symptoms, environment, support at home, time and financial constraints, and lack of patient-centred support and motivation, amongst others [16]. PwMS reported that supplementation and dietary changes were easiest because they were less effortful and had less impact on social or family life. In contrast, initiating new habits that were time consuming, such as meditation and exercise, were challenging for most PwMS. Enablers for adherence to healthy behaviours were identified as experiential learning, peer support, and practical strategies [16]. Future studies to understand how health communication may assist to overcome barriers and incorporate enablers for adopting and maintaining changes in these behaviours is required.

The generalisability of our findings is limited as attrition was significant (61.4%) at 5 years, although this may be expected in long-term online research. The reasons for loss to follow-up, which includes a change in email address and death, were not captured. We queried only a specific source of lifestyle information for pwMS; other sources of lifestyle information may have been accessed by participants and contributed to their behaviour. Given the previously reported healthy participant bias in HOLISM participants [18] and cohort characteristics by missingness at the 5-year review, a larger and more diverse population of pwMS may provide a better understanding of associations between behavioural change and health information engagement. It is possible that changes in lifestyle occurred earlier than the 5-year follow-up, with some participants returning to previous behaviours; the temporality of healthy behaviour and engagement is difficult to establish. Additionally, self-reported adherence to healthy behaviours may be overestimated with social desirability to meet guidelines [48,49]; validated tools deriving a score for healthy behaviours may offer better assessments [50]. Nevertheless, our findings demonstrate that engagement with health information is associated with adoption and long-term maintenance of healthy lifestyle behaviours.

## 5. Conclusions

Lifestyle interventions are increasingly popular for self-management of long-term health conditions. To facilitate knowledge translation and therefore allow informed decision-making for self-management, effective health communication is critical. Our findings suggest that engagement with reading resources combined with face-to-face interactive educational workshops that demonstrate implementation of behaviours in daily life may be optimal for long-term adoption and maintenance of dietary behavioural change in pwMS. This may assist to develop and implement health information resources for self-management through lifestyle modifications.

## Figures and Tables

**Table 1:** Reference and outcome groups for lifestyle behaviour change from baseline to the 5-year review.

	Adoption (Baseline to 5 Year) *		Maintenance (Baseline to 5 Year) *	
Behaviour	Healthy	Unhealthy	Healthy	Unhealthy
Reference	No → No	Yes → Yes	Yes → No	No → Yes
Outcome	No → Yes	Yes → No	Yes → Yes	No → No

* Adoption and maintenance were categorized as binary variables, with the reference category being no adoption/maintenance and the outcome category being adoption/maintenance.

**Table 2:** Cohort characteristics by engagement with resources at baseline.

	Baseline Resource Engagement		
	None	Medium	High
	*n* = 205	*n* = 605	*n* = 142
	Frequency (percentage)		
**Sex**			
Male	24 (11.7%)	111 (18.3%)	28 (19.7%)
Female	181 (88.3%)	494 (81.7%) ^†^	114 (80.3%) ^†^
**Education**			
No/basic schooling	48 (23.6%)	107 (17.8%)	25 (17.6%)
Vocational training	35 (17.2%)	78 (13.0%)	13 (9.2%)
University degree	120 (59.1%)	416 (69.2%) ^†^	104 (73.2%)
**Employment**			
Unemployed	94 (46.1%)	338 (56.0%)	58 (41.1%)
Paid employment	47 (23.0%)	143 (23.7%)	57 (40.4%) ^†^
Retired	63 (30.9%)	123 (20.4%) ^†^	26 (18.4%)
**Marital status**			
Single	23 (11.5%)	81 (13.5%)	13 (9.2%)
Married/partnered	150 (75.0%)	473 (78.8%)	120 (85.1%)
Separated/divorced/widowed	27 (13.5%)	46 (7.7%) ^†^	8 (5.7%)
**MS type**			
Benign/RRMS	131 (75.7%)	435 (81.6%)	102 (81.0%)
SPMS/PPMS/PRMS	42 (24.3%)	98 (18.4%)	24 (19.0%)
**Disability (PDDS)**			
Normal/mild	80 (45.7%)	401 (66.3%)	88 (62.0%)
Moderate	79 (45.1%)	168 (27.8%) ^‡^	46 (32.4%) ^†^
Severe	16 (9.1%)	36 (6.0%)	8 (5.6%)
**Clinical fatigue (FSS>5)**			
No	43 (27.0%)	252 (43.8%)	73 (54.1%)
Yes	116 (73.0%)	324 (56.3%) ^‡^	62 (45.9%) ^‡^
**BMI category**			
Underweight/normal	96 (46.8%)	416 (68.8%)	95 (66.9%)
Overweight	56 (27.3%)	110 (18.2%) ^†^	34 (23.9%)
Obese	53 (25.9%)	79 (13.1%) ^‡^	13 (9.2%) ^‡^
	Mean (SD; range)		
**Age**	46.7 (9.7; 20.4–66.2)	45.1 (10.4; 19.1–70.4)	48.3 (10.8; 23.2–78.5)
	Median (interquartile range)		
**Duration since onset (years)**	15.4 (8.5–25.4)	10.4 (5.3–17.4) ^‡^	13.4 (6.5–22.4)
**PDDS**	3 (1–4)	1 (0–3) ^‡^	1 (0–3) ^†^

Differences of variables by baseline health communication engagement were assessed using log binomial regression. ^†^ *p* < 0.05 for differences between medium/high engagers with non-engagers (reference). ^‡^ *p* < 0.001 for differences between medium/high engagers with non-engagers (reference). Abbreviations: BMI = Body mass index; PDDS = Patient-determined disease steps; PPMS = Primary progressive multiple sclerosis; PRMS = Progressive-relapsing multiple sclerosis; RRMS = Relapsing-remitting multiple sclerosis.

**Table 3:** Baseline associations of lifestyle with resource engagement.

Baseline Lifestyle						
	No at Baseline	Yes at Baseline	Unadjusted		Adjusted *	
	*n*/*N* (Percentage)	*n*/*N* (Percentage)	PR Est.	(95%CI)	PR Est.	(95%CI)
**Taking omega-3 supplement?**						
**Engagement**						
None	114/281 (40.6%)	91/671 (13.6%)	Ref		Ref	
Medium	146/281 (52.0%)	459/671 (68.4%)	**1.71**	**(1.46, 2.01)**	**1.42**	**(1.21, 1.66)**
High	21/281 (7.5%)	121/671 (18.0%)	**1.92**	**(1.62, 2.27)**	**1.56**	**(1.32, 1.83)**
			***PTREND***	***< 0.001***	***PTREND***	***< 0.001***
**Taking ≥ 5000 IU/d vitamin D supplement?**						
**Engagement**						
None	155/460 (33.7%)	49/480 (10.2%)	Ref		Ref	
Medium	275/460 (59.8%)	322/480 (67.1%)	**2.25**	**(1.74, 2.90)**	**1.85**	**(1.44, 2.37)**
High	30/460 (6.5%)	109/480 (22.7%)	**3.26**	**(2.52, 4.23)**	**2.70**	**(2.09, 3.49)**
			***PTREND***	***< 0.001***	***PTREND***	***< 0.001***
**Seeking sun exposure to raise vitamin D level?**						
**Engagement**						
None	86/262 (32.8%)	86/653 (13.2%)	Ref		Ref	
Medium	149/262 (56.9%)	454/653 (69.5%)	**1.51**	**(1.29, 1.76)**	**1.49**	**(1.26, 1.76)**
High	27/262 (10.3%)	113/653 (17.3%)	**1.61**	**(1.36, 1.91)**	**1.57**	**(1.31, 1.89)**
			***PTREND***	***< 0.001***	***PTREND***	***< 0.001***
**Meditating ≥ 10 min/week?**						
**Engagement**						
None	147/677 (21.7%)	22/232 (9.5%)	Ref		Ref	
Medium	468/677 (69.1%)	131/232 (56.5%)	**1.68**	**(1.11, 2.55)**	**1.65**	**(1.09, 2.48)**
High	62/677 (9.2%)	79/232 (34.1%)	**4.30**	**(2.84, 6.53)**	**3.94**	**(2.60, 5.97)**
			***PTREND***	***< 0.001***	***PTREND***	***< 0.001***
**Vigorous physical activity ≥ 30 min/d ≥ 3 times/week?**						
**Engagement**						
None	166/769 (21.6%)	39/182 (21.4%)	Ref		Ref	
Medium	495/769 (64.4%)	109/182 (59.9%)	0.95	(0.68, 1.32)	**0.58**	**(0.42, 0.80)**
High	108/769 (14.0%)	34/182 (18.7%)	1.26	(0.84, 1.89)	0.76	(0.51, 1.14)
			*PTREND*	*= 0.35*	*PTREND*	*= 0.23*
**Current smoker?**						
**Engagement**						
None	149/848 (17.6%)	28/73 (38.4%)	Ref		Ref	
Medium	560/848 (66.0%)	43/73 (58.9%)	**0.45**	**(0.29, 0.70)**	**0.53**	**(0.32, 0.87)**
High	139/848 (16.4%)	2/73 (2.7%)	**0.09**	**(0.02, 0.37)**	**0.12**	**(0.03, 0.54)**
			***PTREND***	***< 0.001***	***PTREND***	***< 0.001***
**Consuming dairy?**						
**Engagement**						
None	30/435 (6.9%)	146/482 (30.3%)	Ref		Ref	
Medium	287/435 (66.0%)	313/482 (64.9%)	**0.63**	**(0.57, 0.70)**	**0.69**	**(0.62, 0.78)**
High	118/435 (27.1%)	23/482 (4.8%)	**0.20**	**(0.13, 0.29)**	**0.20**	**(0.13, 0.31)**
			***PTREND***	***< 0.001***	***PTREND***	***< 0.001***
**Consuming meat?**						
**Engagement**						
None	19/405 (4.7%)	158/519 (30.4%)	Ref		Ref	
Medium	267/405 (65.9%)	338/519 (65.1%)	**0.63**	**(0.57, 0.68)**	**0.67**	**(0.61, 0.74)**
High	119/405 (29.4%)	23/519 (4.4%)	**0.18**	**(0.12, 0.26)**	**0.19**	**(0.13, 0.28)**
			***PTREND***	***< 0.001***	***PTREND***	***< 0.001***

Note: Associations with *p* < 0.050 in bold. * Adjusted for baseline age, sex, education, employment, clinical fatigue, disability, and duration since onset.

**Table 4:** Associations of adopting lifestyle behaviours at five years with baseline health communication modes.

**Lifestyle Adoption**						
	**No at Baseline and 5-yr**	**No at Baseline; Yes at 5-yr**	**Unadjusted**		Adjusted *	
	***n*/*N* (Percentage)**	***n*/*N* (Percentage)**	**RR Est.**	**(95%CI)**	**RR Est.**	**(95%CI)**
**Taking omega-3 supplement?**						
**Engagement**						
None	80/188 (42.6%)	34/93 (36.6%)	Ref		Ref	
Medium	97/188 (51.6%)	49/93 (52.7%)	1.13	(0.78, 1.62)	**1.70**	**(1.02, 2.84)**
High	11/188 (5.9%)	10/93 (10.8%)	1.60	(0.94, 2.71)	**2.50**	**(1.29, 4.85)**
			*PTREND*	*= 0.16*	***PTREND***	***= 0.005***
**Taking ≥ 5000 IU/d vitamin D supplement?**						
**Engagement**						
None	86/248 (34.7%)	66/205 (32.2%)	Ref		Ref	
Medium	144/248 (58.1%)	128/205 (62.4%)	1.08	(0.87, 1.35)	1.19	(0.90, 1.57)
High	18/248 (7.3%)	11/205 (5.4%)	0.87	(0.53, 1.44)	0.98	(0.57, 1.67)
			*PTREND*	*= 0.91*	*PTREND*	*= 0.55*
**Seeking sun exposure to raise vitamin D level?**						
**Engagement**						
None	66/187 (35.3%)	19/70 (27.1%)	Ref		Ref	
Medium	100/187 (53.5%)	46/70 (65.7%)	1.41	(0.89, 2.24)	1.19	(0.73, 1.94)
High	21/187 (11.2%)	5/70 (7.1%)	0.86	(0.36, 2.08)	1.04	(0.44, 2.42)
			*PTREND*	*= 0.62*	*PTREND*	*= 0.67*
**Meditating ≥ 10 min/week?**						
**Engagement**						
None	116/529 (21.9%)	19/93 (20.4%)	Ref		Ref	
Medium	368/529 (69.6%)	67/93 (72.0%)	1.09	(0.68, 1.75)	1.14	(0.69, 1.89)
High	45/529 (8.5%)	7/93 (7.5%)	0.96	(0.43, 2.14)	1.09	(0.47, 2.50)
			*PTREND*	*= 0.93*	*PTREND*	*= 0.72*
**Vigorous physical activity ≥ 30 min/d for ≥ 3 times/week?**						
**Engagement**						
None	152/651 (23.3%)	14/117 (12.0%)	Ref		Ref	
Medium	412/651 (63.3%)	83/117 (70.9%)	**1.99**	**(1.16, 3.41)**	**2.16**	**(1.03, 4.55)**
High	87/651 (13.4%)	20/117 (17.1%)	**2.22**	**(1.17, 4.20)**	**2.71**	**(1.22, 6.01)**
			***PTREND***	***= 0.006***	***PTREND***	***= 0.007***
**Lifestyle adoption**						
	**Yes at baseline and 5-yr**	**Yes at baseline; No at 5-yr**	**Unadjusted**		Adjusted *	
	*n*/*N* (Percentage)	*n*/*N* (Percentage)	RR Est.	(95%CI)	RR Est.	(95%CI)
**Current smoker?**						
**Engagement**						
None	16/38 (42.1%)	12/35 (34.3%)	Ref		Ref	
Medium	21/38 (55.3%)	22/35 (62.9%)	1.19	(0.71, 2.01)	1.46	(0.83, 2.56)
High	1/38 (2.6%)	1/35 (2.9%)	1.17	(0.27, 5.03)	2.29	(0.58, 9.04)
			*PTREND*	*= 0.52*	*PTREND*	*= 0.14*
**Consuming dairy?**						
**Engagement**						
None	135/399 (33.8%)	10/81 (12.4%)	Ref		Ref	
Medium	248/399 (62.2%)	64/81 (79.0%)	**2.97**	**(1.57, 5.63)**	**3.98**	**(1.85, 8.56)**
High	16/399 (4.0%)	7/81 (8.6%)	**4.41**	**(1.87, 10.44)**	**4.94**	**(1.90, 12.88)**
			***PTREND***	***< 0.001***	***PTREND***	***< 0.001***
**Consuming meat?**						
**Engagement**						
None	150/453 (33.1%)	7/64 (10.9%)	Ref		Ref	
Medium	283/453 (62.5%)	54/64 (84.4%)	**3.59**	**(1.67, 7.72)**	**3.42**	**(1.47, 7.91)**
High	20/453 (4.4%)	3/64 (4.7%)	2.93	(0.81, 10.53)	3.01	(0.73, 12.40)
			***PTREND***	***< 0.001***	***PTREND***	***= 0.002***

Note: Associations with *p* < 0.050 in bold. * Adjusted for baseline age, sex, education, employment, clinical fatigue, disability, and duration since onset.

**Table 5:** Associations of maintaining lifestyle behaviours at five years with baseline health communication modes.

**Lifestyle Maintenance**						
	**Yes at Baseline; No at 5-yr**	**Yes at Baseline & 5-yr**	**Unadjusted**		Adjusted *	
	***n*/*N* (Percentage)**	***n*/*N* (Percentage)**	**RR Est.**	**(95%CI)**	**RR Est.**	**(95%CI)**
**Taking omega-3 supplement?**						
**Engagement**						
None	29/178 (16.3%)	62/493 (12.6%)	Ref		Ref	
Medium	130/178 (73.0%)	329/493 (66.7%)	1.05	(0.90, 1.22)	1.08	(0.92, 1.26)
High	19/178 (10.7%)	102/493 (20.7%)	**1.24**	**(1.05, 1.45)**	**1.26**	**(1.06, 1.49)**
			***PTREND***	***= 0.003***	***PTREND***	***= 0.003***
**Taking ≥ 5000 IU/d vitamin D supplement?**						
**Engagement**						
None	13/99 (13.1%)	35/372 (9.4%)	Ref		Ref	
Medium	77/99 (77.8%)	239/372 (64.2%)	1.04	(0.86, 1.25)	1.05	(0.86, 1.29)
High	9/99 (9.1%)	98/372 (26.3%)	**1.26**	**(1.05, 1.51)**	**1.26**	**(1.04, 1.54)**
			***PTREND***	***< 0.001***	***PTREND***	***= 0.001***
**Seeking sun exposure to raise vitamin D level?**						
**Engagement**						
None	22/168 (13.1%)	63/470 (13.4%)	Ref		Ref	
Medium	121/168 (72.0%)	323/470 (68.7%)	0.98	(0.85, 1.13)	0.98	(0.84, 1.13)
High	25/168 (14.9%)	84/470 (17.9%)	1.04	(0.88, 1.22)	0.99	(0.83, 1.18)
			*PTREND*	*= 0.58*	*PTREND*	*= 0.97*
**Meditating ≥ 10 min/week?**						
**Engagement**						
None	8/81 (9.9%)	10/103 (9.7%)	Ref		Ref	
Medium	47/81 (58.0%)	52/103 (50.5%)	0.95	(0.60, 1.49)	0.97	(0.61, 1.55)
High	26/81 (32.1%)	41/103 (39.8%)	1.10	(0.70, 1.74)	1.10	(0.69, 1.75)
			*PTREND*	*= 0.40*	*PTREND*	*= 0.51*
**Vigorous physical activity ≥ 30 min/d for ≥ 3 times/week?**						
**Engagement**						
None	18/83 (21.7%)	21/99 (21.2%)	Ref		Ref	
Medium	46/83 (55.4%)	63/99 (63.6%)	1.08	(0.78, 1.51)	0.92	(0.63, 1.34)
High	19/83 (22.9%)	15/99 (15.2%)	0.82	(0.51, 1.32)	0.67	(0.41, 1.10)
			*PTREND*	*= 0.45*	*PTREND*	*= 0.10*
**Lifestyle maintenance**						
	**No at baseline; Yes at 5-yr**	**No at baseline and 5-yr**	**Unadjusted**		Adjusted *	
	*n*/*N* (Percentage)	*n*/*N* (Percentage)	RR Est.	(95%CI)	RR Est.	(95%CI)
**Current smoker?**						
**Engagement**						
None	6/16 (37.5%)	142/831 (17.1%)	Ref		Ref	
Medium	9/16 (56.3%)	551/831 (66.3%)	1.03	(0.99, 1.06)	1.02	(0.99, 1.06)
High	1/16 (6.3%)	138/831 (16.6%)	1.03	(1.00, 1.07)	1.03	(0.99, 1.07)
			*PTREND*	*= 0.062*	*PTREND*	*= 0.13*
**Consuming dairy?**						
**Engagement**						
None	13/92 (14.1%)	17/339 (5.0%)	Ref		Ref	
Medium	59/92 (64.1%)	225/339 (66.4%)	**1.40**	**(1.02, 1.92)**	1.39	(0.98, 1.97)
High	20/92 (21.7%)	97/339 (28.6%)	**1.46**	**(1.06, 2.02)**	**1.47**	**(1.03, 2.10)**
			***PTREND***	***= 0.019***	***PTREND***	***= 0.023***
**Consuming meat?**						
**Engagement**						
None	5/75 (6.7%)	14/328 (4.3%)	Ref		Ref	
Medium	55/75 (73.3%)	212/328 (64.6%)	1.08	(0.82, 1.42)	1.04	(0.79, 1.36)
High	15/75 (20.0%)	102/328 (31.1%)	1.18	(0.90, 1.56)	1.13	(0.86, 1.49)
			***PTREND***	***= 0.039***	*PTREND*	*= 0.076*

Note: Associations with *p*-value < 0.050 are shown in bold. * Adjusted for baseline age, sex, education, employment, clinical fatigue, disability, and duration since onset.

## Supplementary Materials

The following are available online at https://www.mdpi.com/1660-4601/17/16/5935/s1, Supplementary Table S1: Sociodemographic and lifestyle characteristics of HOLISM main cohort by missingness at 5-year review; Table S2: Associations of baseline resource engagement with lifestyle behaviours at five-year.
