# Supplementary material for: Greater Engagement with Health Information Is Associated with Adoption and Maintenance of Healthy Lifestyle Behaviours in People with MS
Source: Int J Environ Res Public Health. 2020 Aug 15;17(16):5935. doi: 10.3390/ijerph17165935 (PMC7460057; doi:10.3390/ijerph17165935)
Supplement: Supplementary file 1 [file ijerph-17-05935-s001.pdf]

Table S1. Sociodemographic and lifestyle characteristics of HOLISM main cohort by missingness at 5-year review.

Cohort Characteristic	Baseline, Completed 5-Year		Baseline, not Completed 5-Year		5-Year	
	n = 952		*n* = 1514		*n* = 952	
	Frequency (percentage)					
**Sex**						
Male	163	(17.1%)	252	(17.8%)	163	(17.1%)
Female	789	(82.9%)	1161	(82.2%)	789	(82.9%)
**Education**						
No/basic schooling	180	(19.0%)	423	(28.1%)	126	(13.3%)
Vocational training	126	(13.3%)	270	(17.9%)	146	(15.5%)
University degree	640	(67.7%)	813	(54.0%)‡	672	(71.2%)
**Employment**						
Unemployed	490	(51.6%)	767	(51.0%)	145	(15.4%)
Paid employment	247	(26.0%)	283	(18.8%) †	500	(53.1%)
Retired	212	(22.3%)	455	(30.2%) †	297	(31.5%)
**Marital status**						
Single	117	(12.4%)	240	(16.2%)	101	(10.7%)
Married/partnered	743	(79.0%)	1044	(70.3%) †	725	(77.0%)
Separated/divorced/widowed	81	(8.6%)	202	(13.6%)	115	(12.2%)
**MS type**						
Benign/RRMS	668	(80.3%)	923	(73.4%)	668	(80.3%)
SPMS/PPMS/PRMS	164	(19.7%)	334	(26.6%) ‡	164	(19.7%)

**Disability (PDDS)**						
Normal/mild	569	(61.7%)	694	(50.4%)	558	(58.6%)
Moderate	293	(31.8%)	502	(36.5%) ‡	262	(27.5%)
Severe	60	(6.5%)	180	(13.1%) ‡	132	(13.9%)
**Clinical fatigue (FSS > 5)**						
No	368	(42.3%)	367	(28.9%)	382	(41.6%)
Yes	502	(57.7%)	901	(71.1%) ‡	536	(58.4%)
**BMI category**						
Underweight/normal	607	(63.8%)	794	(53.3%)	564	(59.2%)
Overweight	200	(21.0%)	356	(23.9%) †	223	(23.4%)
Obese	145	(15.2%)	340	(22.8%) ‡	165	(17.3%)
**Resource engagement**						
None	205	(21.5%)	590	(39.0%)	122	(12.8%)
Medium	605	(63.6%)	818	(54.0%) ‡	609	(64.0%)
High	142	(14.9%)	106	(7.0%) ‡	221	(23.2%)
**Taking omega-3 supplements?**						
No	281	(29.5%)	717	(47.4%)	366	(38.4%)
Yes	671	(70.5%)	797	(52.6%) ‡	586	(61.6%)
**Taking ≥ 5000 IU/d vitamin D supplement?**						
No	460	(48.9%)	926	(62.6%)	351	(37.5%)
Yes	480	(51.1%)	554	(37.4%) ‡	585	(62.5%)
**Seeking sun exposure to raise vitamin D level?**						
No	262	(28.6%)	485	(36.2%)	363	(38.9%)

Yes	653	(71.4%)	854	(63.8%) ‡	569	(61.1%)
**Meditating ≥ 10 min/week?**						
No	677	(74.5%)	1037	(79.1%)	640	(75.7%)
Yes	232	(25.5%)	274	(20.9%) †	205	(24.3%)
**Vigorous physical activity ≥ 30 min/d ≥ 3 times/week?**						
No	769	(80.9%)	1301	(85.9%)	734	(77.2%)
Yes	182	(19.1%)	213	(14.1%) †	217	(22.8%)
**Smoking status?**						
Non-/Ex- smokers	848	(92.1%)	1159	(84.8%)	896	(94.2%)
Current smokers	73	(7.9%)	208	(15.2%) ‡	55	(5.8%)
**Consuming meat?**						
No	405	(43.8%)	356	(26.0%)	400	(42.2%)
Yes	519	(56.2%)	1014	(74.0%) ‡	548	(57.8%)
**Consuming dairy?**						
No	435	(47.4%)	427	(31.4%)	431	(45.6%)
Yes	482	(52.6%)	933	(68.6%) ‡	514	(54.4%)
			Mean (SD; range)			
**Age, years**	45.9	(10.4; 19.1–79.0)	45.6	(10.5; 18.0–75.3)	50.9	(10.4; 23.8–84.1)
			Median (interquartile range)			
**Duration since onset, years**	11.5	(5.5–19.5)	12.3	(6.4–20.6)	16.5	(10.5–24.6)
**PDDS**	1.0	(0.0–3.0)	2.0	(0.0–4.0) ‡	1.0	(0.0–4.0)

Note: Associations of baseline variables with missing 5-year review were assessed using log binomial regression. † $p < 0.05$ for associations with missingness at 5-year review. ‡ $p < 0.001$ for associations with missingness at 5-year review**.** Abbreviations: BMI = Body mass index; PDDS = Patient determined disease steps; PPMS = Primary progressive multiple sclerosis; PRMS = Progressive-relapsing multiple sclerosis; RRMS = Relapsing-remitting multiple sclerosis.

Table S2. Associations of baseline resource engagement with lifestyle behaviours at five-year.

	5-Year Lifestyle					
	**No at 5-year**	**Yes at 5-year**	**Unadjusted**		**Adjusted ***	
	***n/N* (Percentage)**	***n/N* (Percentage)**	**RR Est.**	**(95%CI)**	**RR Est.**	**(95%CI)**
**Taking omega-3 supplement?**						
**Engagement**						
None	109/366 (29.8%)	96/586 (16.4%)	Ref		Ref	
Medium	227/366 (62.0%)	378/586 (64.5%)	**1.33**	**(1.14, 1.56)**	**1.39**	**(1.15, 1.67)**
High	30/366 (8.2%)	112/586 (19.1%)	**1.68**	**(1.42, 1.99)**	**1.72**	**(1.42, 2.09)**
			PTREND	***< 0.001***	***PTREND***	***< 0.001***
**Taking ≥ 5000 IU/d vitamin D supplement?**						
**Engagement**						
None	100/351 (28.5%)	101/585 (17.3%)	Ref		Ref	
Medium	224/351 (63.8%)	372/585 (63.6%)	**1.24**	**(1.07, 1.44)**	**1.29**	**(1.08, 1.54)**
High	27/351 (7.7%)	112/585 (19.1%)	**1.60**	**(1.37, 1.88)**	**1.66**	**(1.37, 2.00)**
			PTREND	***< 0.001***	***PTREND***	***< 0.001***
**Seeking sun exposure to raise vitamin D level?**						
**Engagement**						
None	96/363 (26.4%)	107/569 (18.8%)	Ref		Ref	
Medium	221/363 (60.9%)	371/569 (65.2%)	**1.19**	**(1.03, 1.37)**	**1.26**	**(1.05, 1.50)**
High	46/363 (12.7%)	91/569 (16.0%)	**1.26**	**(1.06, 1.50)**	**1.30**	**(1.06, 1.60)**
			PTREND	***= 0.007***	***PTREND***	***= 0.010***
**Meditating ≥ 10 min/week?**						
**Engagement**						
None	151/640 (23.6%)	36/205 (17.6%)	Ref		Ref	
Medium	418/640 (65.3%)	121/205 (59.0%)	1.17	(0.84, 1.63)	1.21	(0.83, 1.76)
High	71/640 (11.1%)	48/205 (23.4%)	**2.10**	**(1.45, 3.02)**	**2.03**	**(1.36, 3.05)**
			PTREND	***< 0.001***	***PTREND***	***= 0.001***
**Vigorous physical activity ≥ 30 min/d for ≥ 3 times/week?**						
**Engagement**						
None	170/734 (23.2%)	35/217 (16.1%)	Ref		Ref	
Medium	458/734 (62.4%)	147/217 (67.7%)	**1.42**	**(1.02, 1.99)**	1.10	(0.76, 1.59)
High	106/734 (14.4%)	35/217 (16.1%)	1.45	(0.96, 2.21)	1.10	(0.71, 1.71)
			PTREND	*= 0.050*	*PTREND*	*= 0.696*
**Current smoker?**						
**Engagement**						
None	181/896 (20.2%)	23/55 (41.8%)	Ref		Ref	
Medium	575/896 (64.2%)	30/55 (54.6%)	**0.44**	**(0.26, 0.74)**	**0.41**	**(0.22, 0.75)**
High	140/896 (15.6%)	2/55 (3.6%)	**0.12**	**(0.03, 0.52)**	**0.16**	**(0.04, 0.68)**
			PTREND	***< 0.001***	***PTREND***	***< 0.001***
**Consuming dairy?**						

**Engagement**						
None	34/431 (7.9%)	169/514 (32.9%)	Ref		Ref	
Medium	292/431 (67.8%)	309/514 (60.1%)	**0.62**	**(0.56, 0.68)**	**0.64**	**(0.57, 0.71)**
High	105/431 (24.4%)	36/514 (7.0%)	**0.31**	**(0.23, 0.41)**	**0.31**	**(0.23, 0.42)**
			PTREND	***< 0.001***	***PTREND***	***< 0.001***
			**Consuming meat?**			
**Engagement**						
None	29/400 (7.3%)	175/548 (31.9%)	Ref		Ref	
Medium	266/400 (66.5%)	338/548 (61.7%)	**0.65**	**(0.60, 0.71)**	**0.68**	**(0.62, 0.76)**
High	105/400 (26.3%)	35/548 (6.4%)	**0.29**	**(0.22, 0.39)**	**0.29**	**(0.21, 0.40)**
			PTREND	***< 0.001***	***PTREND***	***< 0.001***

Note: Associations with p-value < 0.050 are shown in bold. * Adjusted for baseline age, sex, education, employment, clinical fatigue, disability, and duration since onset.
